# Double sperm cloning (DSC) is a promising strategy in mammalian genetic engineering and stem cell research

**DOI:** 10.1186/s13287-020-01907-0

**Published:** 2020-09-07

**Authors:** Zhi-ping Zhang, Jun-tao Zhang, Shu-cheng Huang, Xiu-yuan He, Li-xin Deng

**Affiliations:** College of Veterinary Medicine, Henan Agricultural University, Zhengzhou, 450046 China

**Keywords:** Embryonic stem cell, Double sperm cloning, Regenerative medicine, Reprogramming

## Abstract

Embryonic stem cells (ESCs) derived from somatic cell nuclear transfer (SCNT) and induced pluripotent stem cells (iPSCs) are promising tools for meeting the personalized requirements of regenerative medicine. However, some obstacles need to be overcome before clinical trials can be undertaken. First, donor cells vary, and the reprogramming procedures are diverse, so standardization is a great obstacle regarding SCNT and iPSCs. Second, somatic cells derived from a patient may carry mitochondrial DNA mutations and exhibit telomere instability with aging or disease, and SCNT-ESCs and iPSCs retain the epigenetic memory or epigenetic modification errors. Third, reprogramming efficiency has remained low. Therefore, in addition to improving their success rate, other alternatives for producing ESCs should be explored. Producing androgenetic diploid embryos could be an outstanding strategy; androgenic diploid embryos are produced through double sperm cloning (DSC), in which two capacitated sperms (XY or XX, sorted by flow cytometer) are injected into a denucleated oocyte by intracytoplasmic sperm injection (ICSI) to reconstruct embryo and derive DSC-ESCs. This process could avoid some potential issues, such as mitochondrial interference, telomere shortening, and somatic epigenetic memory, all of which accompany somatic donor cells. Oocytes are naturally activated by sperm, which is unlike the artificial activation that occurs in SCNT. The procedure is simple and practical and can be easily standardized. In addition, DSC-ESCs can overcome ethical concerns and resolve immunological response matching with sperm providers. Certainly, some challenges must be faced regarding imprinted genes, epigenetics, X chromosome inactivation, and dosage compensation. In mice, DSC-ESCs have been produced and have shown excellent differentiation ability. Therefore, the many advantages of DSC make the study of this process worthwhile for regenerative medicine and animal breeding.

## Introduction

Stem cells represent a potential option for the treatment of some major diseases, such as cancer and degenerative diseases. Embryonic stem cells (ESCs) are the best option, representing the “gold standard”; they are obtained from the early mammalian embryo or IVF embryo and possess self-renewal and the capacity to differentiate into a wide variety of cell types, including ectoderm, mesoderm, and endoderm. Since ESCs were first successfully isolated [1–3], scientists have gradually focused on ESC research fields, including regenerative medicine, drug selection, and animal conservation. However, the destruction of embryos raises ethical issues [4]. Furthermore, immune responses of human ESCs have to be faced in clinical use, despite possessing immune-privileged properties [5]. Therefore, innovating alternative ways to obtain ESCs has become highly sought for personalized medicine.

In 1958, a cloned frog was obtained by using the injection of somatic cell nuclei into *Xenopus* oocyte [6], demonstrating that batrachian oocytes were capable of reprogramming somatic cells. When sheep and mice cloned by SCNT were successfully bred, mammalian oocytes were also shown to be able to reprogram somatic donor nuclei to a pluripotent state [7–9]. These great advances evoke the desire for the application of the SCNT technique in animal breeding and even in endangered animal conservation [10]. Reprogramming somatic cells into ESCs by oocytes has also been envisioned as an approach for generating patient-matched SCNT-ESCs for specific therapies and circumventing immune rejection by the host [11, 12]. The genetically totipotent features of SCNT-ESC lines have been verified [13–17].

However, animal cloning is inefficient due to faulty epigenetic reprogramming, which in turn dysregulates gene expression [17–22]. A total of ∼ 9% of the dysregulated genes in SCNT-derived placenta were associated with transcriptomic reprogramming errors [23], which caused cloned animals to have shorter lifespans, most likely due to respiratory failure, hepatic failure, abnormal kidney development, liver steatosis, and large offspring syndrome [20, 24, 25]. All of the developmental abnormalities suggest that reprogramming of donor nuclei may not be fully completed by SCNT [26, 27], disturbing the gene expression patterns [28]. The reconstruction complexity and oocyte dependency of SCNT prompt the exploration of alternative approaches for somatic cell reprogramming. In addition to oocytes, pluripotent cells can dedifferentiate somatic cells by fusion and activate genes (such as the Oct4 gene) that are not expressed in adult cells. Therefore, ESCs or oocytes also contain factors that can confer totipotency or pluripotency to somatic cells [29–32]. Transcription factors, such as Oct3/4 [33, 34], Sox2 [35], and Nanog [36, 37], were confirmed to be effective in the maintenance of pluripotency in both early embryos and ESCs. Some genes, such as Stat3 [38, 39], E-Ras [40], c-Myc [41], Klf4 [42], and β-catenin [43], contributed to the long-term maintenance of the ES cell phenotype and rapid proliferation in vitro. A landmark advance reported that mouse pluripotent stem cells (iPSCs) were directly generated from fibroblast cultures by retroviral transduction of four transcription factors, Oct3/4, Sox2, Klf4, and c-Myc (named the Yamanaka factors) [44]. Subsequently, iPSCs were derived in several species, including humans [45–47] and rhesus monkeys [48], and the iPSCs have normal karyotypes and telomerase activity, express ES cell surface markers and genes, and maintain the developmental potential to differentiate into the three primary germ layers [49]. Similarly, iPSCs were derived from nearly all somatic cell populations, such as keratinocytes [50], neural cells [51, 52], stomach and liver cells [53], melanocytes [54], and lymphocytes [55], via various vectors [56]. To eliminate the risk of genomic integration and insertional mutagenesis, recent methodological improvements, such as treatment with microRNAs [57], synthetic mRNA modified [56], and valproic acid [58] as well as stimulus-triggered acquisition of pluripotency (transient low-pH stressor) [59] and chemically small-molecule compounds [60], enhance the efficiency of reprogramming, reducing genomic modifications. These concentrated gains demonstrate an increasing number of reprogramming strategies, but these achievements also hint that the transcription network governing pluripotency is unclear. Less than 3% of somatic cells give rise to iPSC colonies. iPSCs are heterogeneous and highly diverse compared to ESCs due to epigenetic memory [61, 62] and epigenetic dynamics [63], which exhibit features of incomplete reprogramming and present limitations in disease modeling and personalized medicine [64]. Most iPSCs exhibit particular defects, such as poor quality of differentiation, low growth rate, aberrant transcription, disrupted DNA methylation and chromatin regulation, or chimeric animal contribution [62, 65, 66]. Theoretically, iPSCs can effectively overcome autologous immune rejection, but in contrast to derivatives of ESCs, abnormal gene expression in some cells differentiated from iPSCs can induce a T cell-dependent immune response in syngeneic recipients [67]. iPSCs differed and retained residual DNA methylation patterns typical of parental somatic cells, and the differentiation and epigenetic state of the donor nucleus influence reprogramming efficiency [68]. iPSCs derived from different somatic cell sources show different capacities of reprogramming and differentiation; for example, progenitor cells are more efficient at giving rise to iPSCs than terminally differentiated cells [55, 69]. iPSCs are prone to (epi) genetic instability during in vitro culture [70]. In contrast, the differentiation and methylation characteristics of SCNT cells were more similar to those of classical ESCs than iPSCs [61]. Although iPSC reprogramming is technically simpler, it is less efficient and slower than SCNT and cell fusion [71]. There are strict standards regarding the specificity, efficiency, kinetics, and safety of stem cells for clinical use, so reprogramming, methodological improvements, or fundamental changes in SCNT and iPSCs are considered.

## Definition of double sperm cloning

Compared with iPSCs, SCNT-ESCs bear “fewer abnormalities” and exhibit characteristics that “more closely resemble genuine embryonic stem cell” traits, which may favor their use as therapies in treating particular conditions [72]. Therefore, many challenges must be faced for establishing the standardized procedure of reprogramming somatic cells by SCNT or iPSCs, which may be an elusive topic. Therefore, in addition to paying more attention to improving the reprogramming efficiency in somatic cells by SCNT and iPSCs, we should innovate other reprogramming alternatives that are relatively easy to standardize to fulfill concerns about specificity, efficiency, and safety in clinical use. Here, we define double sperm cloning (DSC), which is based on androgenetic diploid embryos. Strictly defined, DSC involves two capacitated sperm, which are sorted by sex via flow cytometry, that are then injected into denucleated oocytes by intracytoplasmic sperm injection (ICSI). Afterward, the fertilized embryos are cultured to form blastocysts and to derive ESCs. This strategy offers a promising method for regenerative medicine and animal breeding, and it possesses unique superiority to SCNT and iPSC methods. This article mainly illuminates the realization, performance, advantages, and application of DSC, and it highlights the opportunities and challenges compared to SCNT and iPSCs.

## Operability of ESCs derived from double sperm cloning embryos

**Fig. 1:**
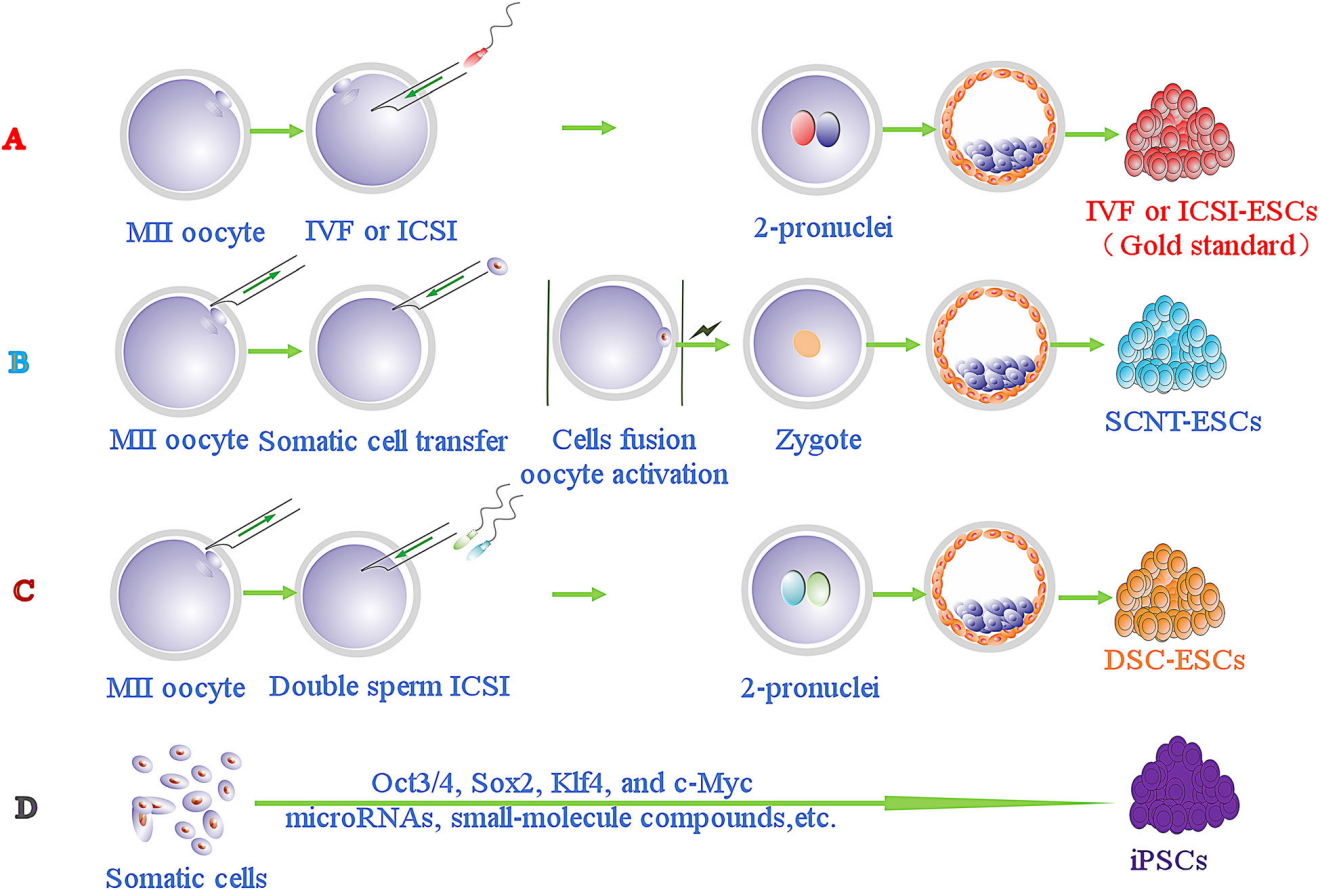
Different reprogramming strategies for deriving embryonic stem cells (ESCs) or pluripotent stem cells. **a** Natural fertilization between sperm and oocytes to develop a blastocyst and generate ESCs by IVF or ICSI. **b** Somatic cell nuclear transfer procedure to isolate SCNT-ESCs, including oocyte and somatic cell fusion, and constructed embryo activation. **c** Double sperm cloning (DSC) by injection of two sperm (X, Y sorted by flow cytometry) into the enucleated oocytes to construct embryos and then isolating DSC-ESCs from blastocysts. **d** Induced pluripotent stem cells (iPSCs) derived from somatic cells by reprogramming factors, such as the Yamanaka factors, microRNAs, and small-molecule compounds

To avoid stability and efficiency issues with somatic cells reprogrammed by SCNT and iPSCs, we used a distinct way to harvest ESCs from the embryo by the DSC method. DSC involves several key steps, including ICSI and the use of very mature enucleated oocytes. For example, metaphase II oocyte cytoplasts are easily enucleated with mechanical [73] or chemically assisted methods [74, 75]. With DSC, the constructed embryos develop into blastocysts and ESCs can be isolated from their inner mass cells (Fig. 1). The strategy not only simulates natural fertilization but also makes good use of established techniques, such as ICSI and oocyte enucleating. It guarantees a normal diploid karyotype of the reconstructed embryo.

The oocyte is the best system for supporting the reprogramming of a homogeneous cell. In fact, natural fertilization is also reprogramming of oocytes to sperm, and reprogramming of somatic nuclei is not as efficient as that of sperm nuclei. Sperm chromatin is epigenetically modified to be adequate for early embryonic development, while somatic nuclei do not have such modifications. Moreover, epigenetic memories encoded in sperm chromatin are transgenerationally inherited, implying unique roles of sperm [76]. In human clinical reproduction, an inspection of pronucleus formation is routine for selecting zygotes with exact two pronuclei after IVF; if zygotes with more than two pronuclei are observed, the embryo must be discarded due to polyspermic fertilization. Therefore, all of these results illustrate that oocytes can simultaneously reprogram two sperm through DSCs. Other procedures refer to sophisticated programs for deriving ESCs from embryos [3, 11]. Hence, the DSC strategy could be used to produce superior ESCs in theory and practice.

## Oocyte activation by sperm factors

In addition to oocyte quality being a prominent part of reprogramming, several steps in SCNT operation, including spindle removal, donor cell fusion, and cytoplast activation, are also critical for cellular reprogramming to blastocyst development [11]. However, natural fertilization involves fewer steps than SCNT does, as the entry of only one sperm triggers oocyte activation, which is critical for the completion of meiosis and the initiation of mitotic divisions. In both sperm and oocytes, activation is also critical for the oocyte cytoplasm to acquire reprogramming and metabolic activity, which is necessary to support subsequent embryo development [77].

Sperm factors are thought to initiate oocyte activation through oscillations in Ca^2+^ at fertilization by sperm-oocyte fusion [78–81], and the demethylation process is facilitated either by a sperm-derived factor or by male pronuclear chromatin composition [82], and calcium plays a pivotal role in this process. The injection of sperm cytosolic extracts into oocytes triggers a prolonged series of Ca^2+^ oscillations similar to those seen at fertilization [83]. In mouse and human ICSI, a prolonged series of Ca^2+^ oscillations is also accompanied by sperm factors. Therefore, oocytes in DSCs are also activated by sperm factors similar to ICSI and IVF. However, somatic cells lack these factors, and oocytes must be activated in other ways, such as chemical activation or electric activation in SCNT. Reactive oxygen species levels and Ca^2+^ oscillations differed between sperm-activated oocytes and parthenogenetically activated oocytes [83, 84]. Therefore, oocyte activation in DSC is more natural and superior for embryo development than what is observed in SCNT. The DSC procedure is simple because it does not require donor cell artificial fusion. Additionally, preprocessing of sperm and somatic cells is different between SCNT and DSC.

## Sperm superiority of reprogramming

Sperm are highly differentiated, transcriptionally inert cells with minimal cytoplasm, and they contain a suite of unique RNAs that are delivered to oocytes. These RNAs are likely involved in many different processes, including genome recognition, early embryonic development, and epigenetic transgenerational inherence [85]. One of the biggest differences between sperm and somatic cells is the fact that somatic cell DNA is wrapped around histones, whereas sperm DNA is tightly packed by protamines, which condenses sperm DNA to one sixth the size of the mitotic chromosomes and carefully protects their DNA [86]. At fertilization, the highly condensed and transcriptionally inert chromatin of the sperm is remodeled into the decondensed and transcriptionally competent chromatin of the male pronucleus [87]. Sperm also carry numerous paternal mRNAs to oocytes at fertilization, facilitating early development [88–90]. Sperm is important for the first cell division and can influence the pattern of embryonic gene expression and even phenotypes of the progeny [91]. Epigenetic marks in sperm are extensive and are correlated with developmental regulators [92]. All of the sperm chromatin features are likely to support embryonic development after fertilization. Somatic chromatin does not have such “fine-tuning” for correct embryonic gene expression. Therefore, embryos generated from SCNT often show abnormal reprogramming events compared to fertilized embryos [76], and the cell cycle state of the donor as well as their level of differentiation may be important determinants of reprogramming efficiency. Scientists compared the differences between iPSCs and ESCs and found persistent donor cell gene expression and epigenetic memories in iPSCs [66, 93–96]; however, sperm express fewer genes and carry fewer epigenetic marks than iPSCs. Therefore, the superiority of DSC is the result of sperm chromatin features, which may be useful in embryo development.

## Mitochondrial features

Mitochondrial DNA (mtDNA) possesses unique properties, including high copy numbers, maternal inheritance, lack of recombination, and high mutation rate. Many mtDNA mutations have been found to be related to aging, neurodegeneration, and tumorigenesis [97–99]. Aged somatic cells might show high susceptibility to nuclear and mitochondrial genome instability [100]. Hypothetically, in reprogrammed somatic cells from patients to generate pluripotent stem cells for therapeutic application, mtDNA mutations of the somatic cell must be evaluated, including analysis of a broad spectrum of degenerative diseases associated with mutations in mtDNA, which are unlikely to be amenable to iPSC-based therapies due to the persistence of the somatic cell mtDNA mutations [101]. Furthermore, mature oocytes contain more than 150, 000 copies of mtDNA, which is at least an order of magnitude greater than the number in most somatic cells, and sperm contain only approximately 100 copies [102]. ICSI performed with mature sperm does not alter the uniparental pattern of inheritance of mtDNA, and mtDNA is selectively degraded through a selective silencing process that occurs early in development [103]. In mice, most of the offspring carried donor cell-derived mtDNA that constituted as much as 13.1% of the total [104]. Therefore, the small amount of mtDNA provided via sperm by DSC cannot disturb embryo development and suggests its safety in clinical use, such as in iPSCs and somatic cells reprogrammed by SCNT.

## Telomere importance

Telomeres are protective end complexes at the end of mammalian chromosomes. Telomere length gradually shortens as stem cells divide to produce differentiated cells, eventually resulting in replicative senescence with aging. Sometimes, the loss of telomeres can lead to potentially maladaptive cellular changes, block cell division, and interfere with tissue replenishment, especially in diseases of human aging and in some aging-related processes [105]. Telomeres and telomerase are the main components of the stem cell “ignition” mechanism for tissue regeneration and provide a way to restrain cancer and delay aging [106]. Telomere lengths are highly correlated with the developmental pluripotency of ESCs. For example, ESCs with long telomeres exhibit authentic developmental pluripotency, as evidenced by the generation of complete ESC-derived offspring as well as germline-competent chimeras [107]. Cells with short telomeres cannot be reprogrammed to generate iPS cells despite their normal proliferation rates [108]. Therefore, telomere length is considered a valuable marker for evaluating stem cell pluripotency. Telomerase is strongly expressed in ESCs and is inactive in most somatic cells. Therefore, telomeres and telomerase must be key factors in somatic reprogramming to stem cells. Telomeric chromatin is remodeled, and telomeres are elongated by telomerase during nuclear reprogramming. For example, telomerase activity and telomere lengths were increased in SCNT-derived blastocysts compared to donor cells [109, 110]. This suggests that oocytes possess a perfect reprogramming cytoplasm for donor cells. Similarly, compared to differentiated cells, iPSCs also have longer telomeres with increasing passages until telomeres reach a length that is characteristic of ES cells [111]. SCNT-mediated reprogramming mitigates telomere dysfunction and mitochondrial defects to a greater extent than iPSC-based reprogramming [112], and ESCs have greater differentiation potential and self-renewal capacity than iPSCs. Telomeres in mammalian male germ cells progressively increase in length from spermatogonia to sperm during spermatogenesis. However, telomerase activity is gradually downregulated during germ cell differentiation from spermatogonia to sperm, and no telomerase activity occurs in the spermatozoa [113]. Therefore, in DSC, two sperm with long telomeres are introduced into oocytes with stronger reprogramming capacity and higher telomerase activity, which is similar to the situation in IVF embryos. Theoretically, we can derive DSC-ESCs with normal telomeres from the DSC-derived blastocyst.

## Storage superiority of sperm

**Fig. 2:**
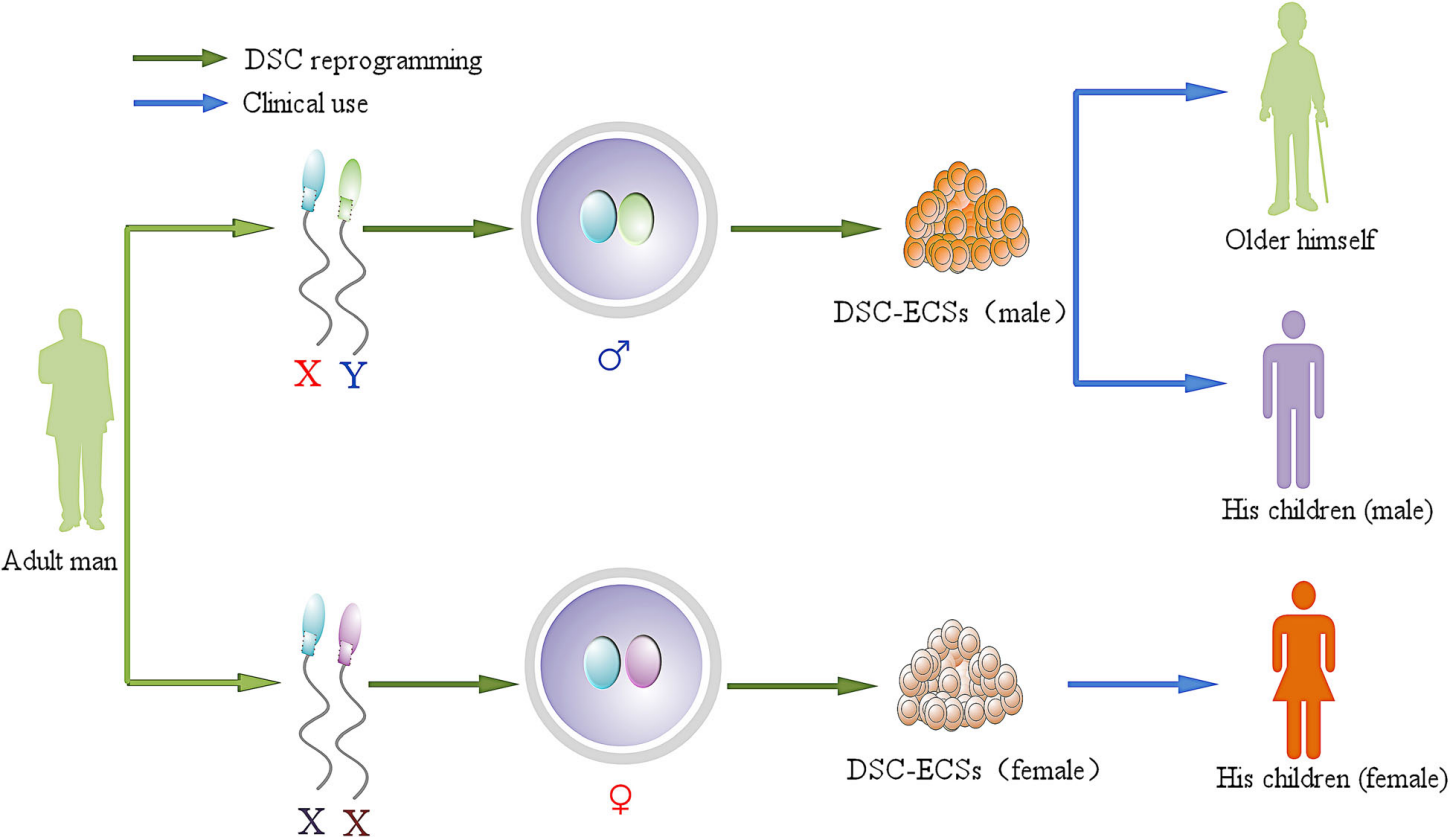
DSC-ESCs are designed for clinical use for autologous transplantation when the donor is aged or develops a disease. They can be considered as alternative means of treatment for the donor’s children when needed according to their sex (XY, XX) because all of the DNA of DSC-ESCs is only from his father

This method may be used to obtain embryos and derive specific DSC-ESCs. So, the sperm bank has a new function. Compared to cell cryopreservation for clinical use, sperm cryopreservation is simple and completely practicable, and its maintenance cost is much lower than that of somatic cells. Theoretically, DSC-ESCs could be used to cure donor disease, and they could also be used to treat his children. Personalized DSC-ESCs can offer two types, XY and XX, for donor and his baby, whose chromosome only comes from their father (Fig. 2).

## Progress of DSC

At present, scientists mainly focus on the establishment of haploid embryonic stem cells [114, 115]. In 2012, Hui Y [116] and Wei Li [117] reported the derivation of androgenetic haploid ES (ahES) cells from androgenetic blastocysts, and they obtained live mice upon injection of ahES cells into MII oocytes. In 2015, Ding C produced androgenetic haploid human embryos by injecting a single spermatozoon into an enucleated human oocyte and established human androgenetic embryonic stem (hAGES) cell lines from androgenetic embryos, which exhibit typical features of human ESCs [118]. The hAGES cells maintained a sperm methylation pattern to a certain extent. The ahES cells could produce viable and fertile progenies after intracytoplasmic injection into mature oocytes [118]. These achievements are similar to what can be achieved with DSC-ESCs. All of these studies show that enucleated oocytes are completely capable of reprogramming one sperm and gaining ahES cells exhibiting typical features of ESCs. In 1984, James McGrath constructed diploid mouse embryos with two male pronuclei by transplantation of pronuclei and found that they could not develop to term. They concluded that a diploid genome derived from only one of the two parental sexes is incapable of supporting complete embryogenesis [118], and imprinted genes showed paternal bias in the placenta but not in the fetus [119]. Kono et al. increased the concentration of sperm use in fertilization with enucleated oocytes, resulting in 35–45% efficiency of fertilizing eggs, which produced heterozygous bispermic androgenones, and 43% of embryos developed to blastocysts [120]. Lagutina et al. reported that 31% of bovine diploid androgenetic embryos (DSC embryos) could develop into blastocysts, which was a rate similar to that of IVF control embryos (35%), and following the transfer of diploid androgenetic embryos, a pregnancy could be established and maintained up to day 28 [121]. In sheep, Matsukawa et al. reported the use of IVF in producing diploid androgenetic embryos resulted in no significant difference in early cleavage and morula, but the blastocyst formation rate was significantly lower. However, diploid androgenetic embryos produced by pronuclear exchange developed to the blastocyst stage at a higher proportion (19%) [122]. Theoretically, two Y chromosome embryos cannot regularly develop due to the lack of the X chromosome; if the “aberrant” androgenetic embryo (YY) can be ruled out, the blastocyst formation rate is higher. Furthermore, the developmental potential of haploid embryos is significantly impaired relative to diploid embryos; Latham et al. discovered subtle alterations in the gene expression in haploid androgenones, such as the lack of repression of the Pgk1 gene, from what is seen in diploid androgenones, which experience X chromosome inactivation [123]. The blastocyst formation rate from androgenetic diploid embryos is higher than that from androgenetic haploid embryos (43% vs 11%, respectively, in mice [120] and 31% vs 1.8%, respectively, in bovines [121]). Therefore, we should derive more DSC blastocysts (androgenetic diploid embryos) for isolating ESCs. In 2009, Teramura et al. established authentic ESCs from androgenetic diploid mouse embryos by IVF from two sperm and a denucleated oocyte by taking advantage of adjusting the sperm concentration and the zona pellucida incision [124], and they induced differentiation of mouse AgESCs and observed derivation of neural cells, cardiomyocytes, and hepatocytes in vitro and found that an embryoid body generated from the cells could engraft in theoretically MHC-matched strains [124]. Dinger et al. also observed that AgESC-derived neural progenitor/stem cells do not differ from normal neural progenitor/stem cells in their self-renewal and neural differentiation potential in mice in vivo and in vitro and exhibited fidelity regarding the expression of six imprinted genes analyzed, though the expression of Ube3a had changed [125]. Therefore, DSC-ESCs is promising.

## Clinical therapy and ethics related to DSC-ESCs

Regardless of the stem cell type, before the cells can be used in regenerative medicine, the safety and standard procedures must be established. For natural embryos, the procedure of establishing ESCs is easy to standardize. Once efficient differentiation protocols for the generation of a target cell lineage are established, ESCs are expected to stably supply large amounts of cell substrates for use in cell-based therapies, which have greater differentiation and replication ability than somatic stem cells. Human ES cell-based products were recently evaluated in clinical trials in the USA. However, human ESCs are generated by the destruction of human embryos, so there are ethical issues [126]. ESCs from IVF embryos are allogeneic to the patients that would receive them for treatment. SCNT-ESCs and iPSCs can be derived from the donors’ cell of a patient; they are autologous cells with less immunogenicity, but they are prone to epigenetic and transcriptional aberrations. These cells acquire genetic modifications in addition to epigenetic modifications, and extensive genetic screening should become a standard procedure to ensure safety for clinical use [127]. The protocols to produce ESCs involve many steps or different methods, as mentioned above, making this process difficult to standardize. Compared to somatic cells reprogrammed by SCNT and iPSCs, the DSC approach produces ESCs with a set of standard procedures, and DSC-ESCs are matched with the patients (Fig. 2). Herein, by clarifying the advantages and disadvantages of the current reprogramming systems, we may be providing an effective strategy for generating clinical-grade cells.

## Animal breeding by DSC

**Fig. 3:**
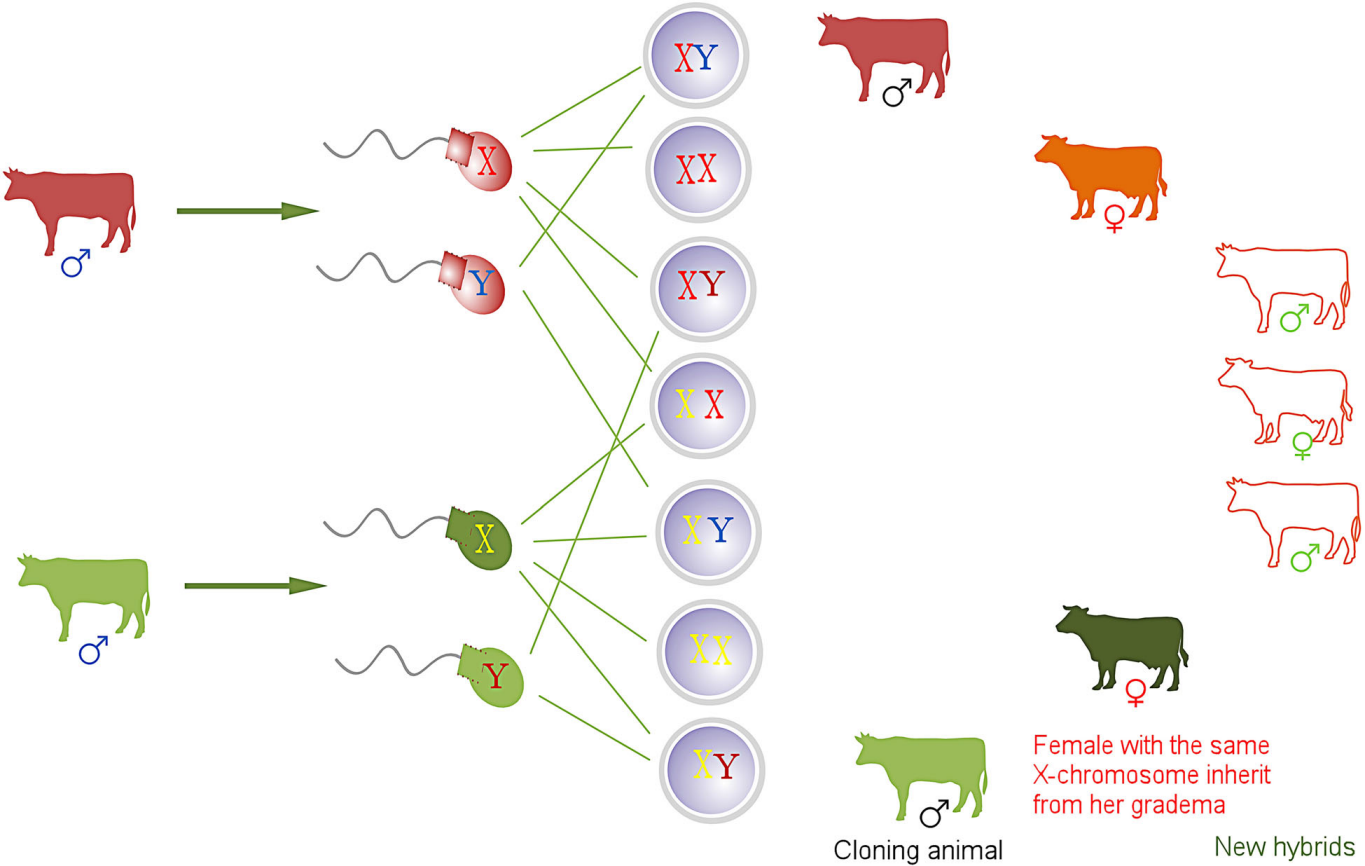
Animal breeding with DSC: cloned animals are bred by DSC (his one XY); female animals inherit the X chromosome from the female animal that parented her parent; breeding new hybrids by DSC

Currently, prevalent livestock animal breeding mainly follows the traditional pattern of progeny testing, which requires many years of breeding unique traits and gains stabilized genetic characteristics following a strict breeding program. With the help of reproductive technology, including artificial insemination and embryo transfer, improved breeds could be popularized for commercial applications. The emergence of animal cloning provides a promising method for breed conservation. In cloned cattle, blood profiles and other indicators of general physiological function, such as growth rate, reproduction, rearing of offspring, and milk production, are all within normal phenotypic ranges [128]. If the challenges of DSC are successfully overcome with increased improvements to the methods, this would provide a great tool for use in animal breeding. Not only can we make use of two sperm (X and Y) from one male individual, similar to SCNT, but we can also apply two X sperm from one male in the production of female offspring. Furthermore, we can establish a new animal breeding system by assembling the sperm of different sexes from diverse breeds (Fig. 3).

## Challenges of DSC, SCNT, and iPSCs

Genomic imprinting occurs via an epigenetic mechanism, which means that some imprinted genes are only expressed from their maternal allele, while others are only expressed from their paternal allele. Approximately 1% of human genes are normally expressed from only the maternally or paternally inherited gene copy [129]. Mammals are biparental diploid organisms, and both maternal and paternal genomes are required for normal development. Genomic imprinting affects several dozen mammalian genes and directs the expression of those genes by DNA methylation, resulting in the expression of either the maternal or paternal allele [130]. DNA methylation memory establishment, maintenance, and erasure are carefully balanced by molecular machinery that is highly conserved among vertebrates [131]. Most imprinted genes contain differentially methylated regions (DMRs) between the maternal and paternal alleles [132]. Disrupted imprinting is associated with significant pathologies [133], such as disruption of the transfer of nutrients [134]. Methylation patterns are reprogrammed genome-wide in embryos and generate cells with broad developmental potential. Epigenetic reprogramming is critical for the imprinting process [135]. The low success rate of SCNT in cloning is largely due to imprinting problems. The percentage of the DMR that was methylated in imprinted genes (XIST and H19) was significantly decreased, and short-lived cloned bovines exhibited more severe aberrant methylation changes in the examined imprinted genes [136]. In certain SCNT-ESC lines, DNA methylation patterns of a paternally imprinted gene, H19, displayed distinct abnormalities and appeared to be very dynamic; maternally imprinted genes, Mest and Peg3, showed relatively stable methylation patterns in ES cells [137]. The altered expression of imprinted genes associated with SCNT is also caused by changes in histone modifications [138]. Similarly, imprinting errors are observed in iPSCs, suggesting that these epigenetic anomalies are related to the reprogramming process and could be directly responsible for the variable phenotypes and low success rates of both cloning and iPS derivation procedures [139]. Certainly, the epigenetic abnormalities detected in iPSCs are not specific to transcription factor-mediated reprogramming [140]. It is difficult to program via SCNT and iPSC induction, and it is also a challenge to cause two sperm from paternal genomes to construct a DSC embryo with the correct reprogramming procedure. Epigenetic studies have demonstrated changes to the DSC procedure that are necessary for mammalian physiology [141]. In DSC embryos, two sperm contain some similar imprinted genes disturbing embryo development. During oocyte reprogramming, imprinted genes could be coordinated to function in accordance with evolutionary conservation [142]. For example, the impact of external environments also results in DNA methylation alterations. Mikhael reported that H19/IGF2 imprinting may be epigenetically stable after reprogramming in cloned horses [143]. Fortunately, DSC embryos can develop blastocysts at a high rate and even establish early pregnancy [120, 121]. Mouse androgenetic diploid ESCs have been established and can differentiate into other cell types [124]. We believe that all of these studies help establish DSC-ESCs as a promising tool for regenerative medicine.

In mammals, epigenetic marks on the X chromosomes are involved in dosage compensation, and transcriptional silencing of one of the two X chromosomes randomly occurs in female cells during late blastocyst development [144]. Incomplete nuclear reprogramming in cloning animals may affect both random and imprinted XCI [145], and many genes on the X chromosome are specifically downregulated [104]. In iPSC reprogramming, mouse iPSCs exhibit X chromosome activation (XCA) of two chromosomes, while there is XCA of only one in humans [146]. Thus, there are different X chromosome statuses in reprogramming. XCI has been shown to vary widely in human female iPSCs and ESCs [147]. Therefore, if we derive human ESCs by DSC, dosage compensation must be accounted for; specifically, the XCI status must be considered in female cells, and in male (XY) DSC-ESCs, X chromosome reactivation must be addressed. The DSC method offers a sex chromosome combination (XX, XY, and YY) for exploring the dosage compensation mechanism.

## Conclusion

Stem cells have the capacity for self-renewal and differentiation into various cell lineages in regenerative medicine. ESCs are the classic representative of stem cells. To meet conditions related to efficient reprogramming, sufficient cell yield, ethical concerns, specificity, and safety, SCNT-ESCs and iPSCs have been generated to produce pluripotent cells with the hope of clinical use. For example, oocytes can reprogram a somatic cell in less than 3 days while retaining an intact genome and epigenome [148], and SCNT-ESCs showed lower chromosome mutation [149]. In addition, accumulating preclinical data also support the safety and efficacy of iPSCs [150]. These emerging technologies motivate new research trends with many exciting achievements, but low reprogramming efficiency and genomic modification steps still restrict the clinical use of these cells [151]. For example, iPSCs always possess somatic-coding mutations [127]. SCNT and iPSCs are not perfect, exhibiting genetic and epigenetic abnormalities and immunogenicity [150]. In addition, their standardization of reprogramming is difficult. Donor cells come from variable differentiated cells, and reprogramming procedure also varies. Donor cells from patients can have mtDNA mutations or exhibit signs of aging. Therefore, we propose a promising alternative, double sperm cloning, to produce ESCs that avoid the abovementioned disadvantages. Theoretically, DSC has many advantages, such as enabling sperm storage, standardization, and specificity as well as maintaining mtDNA quality being a simple procedure and reprogramming process that is similar to natural fertilization. The successful mouse DSC-ESCs strongly support this strategy. However, no live animal was born from DSC, perhaps due to epigenetic modifications. These issues also exist in somatic cells reprogrammed by SCNT or iPSCs. Scientists have ignored such issues; until Dolly the sheep was born, most scientists considered oocyte reprogramming of somatic cells to an embryonic state an impossibility. Most studies have focused on genetics to illuminate why this was impossible. Recently, more studies have been performed on uniparental embryos and have derived uniparental haploid ESCs. DSC deserves more attention and should be studied to achieve goals in medicine and animal breeding.

## Data Availability

Not applicable.

## Abbreviations

ESCs: Embryonic stem cells
SCNT: Somatic cell nuclear transfer
IPSCs: Induced pluripotent stem cells
DSC: Double sperm cloning
ICSI: Intracytoplasmic sperm injection
LIF: Leukemia inhibitory factor
bFGF: Basic fibroblast growth factor
PLCζ: Phospholipase C, zeta
PAWP: Post-acrosomal sheath WW domain-binding protein
mtDNA: Mitochondrial DNA
hAGES: Human androgenetic embryonic stem
DMRs: Differentially methylated regions
XCI: X chromosome inactivation
XCA: X chromosome activation
